# Delayed Thumb Replantation at 24 Hours Following Saw-Cut Amputation at the Base of the Proximal Phalanx: A Case Report and Review of Outcomes

**DOI:** 10.7759/cureus.107242

**Published:** 2026-04-17

**Authors:** Nay Aung Zin, Thura Kyaw, Saw Myat Thu Wynn, Htoo Aung Naing Shwe, Mohamed Arsham Abdul Rasheed, Brang Jar

**Affiliations:** 1 Orthopaedics and Traumatology, Kulhudhuffushi Regional Hospital, Kulhudhuffushi, MDV; 2 Orthopaedics and Trauma, 300 Bedded Orthopaedic Hospital, Mandalay, MMR; 3 Orthopaedics, Hulhumalé Hospital, Hulhumale, MDV; 4 Orthopaedics and Trauma, Himmafushi Health Centre, Himmafushi, MDV; 5 Orthopaedic Surgery, Kulhudhuffushi Regional Hospital, Kulhudhuffushi, MDV; 6 Orthopaedics and Traumatology, University of Medicine 1, Yangon, Yangon, MMR

**Keywords:** delayed replantation, digital amputation, functional outcome, hand reconstruction, hand trauma, ischemia time, microsurgery, proximal phalanx amputation, saw injury, thumb replantation

## Abstract

Thumb amputation is a severe injury that significantly affects hand function. Replantation remains the preferred treatment, particularly in young and active individuals. However, prolonged ischemia time is traditionally considered a limiting factor for successful replantation. We present a case of a 25-year-old right-hand-dominant male who sustained a traumatic saw-cut amputation of the left thumb at the base of the proximal phalanx and underwent successful replantation approximately 24 hours post-injury. Surgical management included skeletal fixation with Kirschner wires, microvascular anastomosis, tendon repair, and nerve coaptation. Serial follow-up demonstrated satisfactory wound healing and progressive functional recovery. At six months, the patient achieved near-normal grip strength, pinch strength, and full thumb opposition. This case highlights that delayed thumb replantation can still result in favorable functional outcomes when appropriate surgical technique and postoperative rehabilitation are employed.

## Introduction

The thumb plays a critical role in hand function, contributing significantly to grip strength, pinch, and opposition, and is essential for performing activities of daily living [1]. Traumatic thumb amputation is therefore a functionally devastating injury, and replantation is considered the treatment of choice whenever feasible, particularly in young and active individuals [2].

The success of digital replantation depends on several factors, including the mechanism and level of injury, ischemia time, and the quality of tissue preservation [3,4]. Traditionally, prolonged ischemia time has been considered a limiting factor, with recommended thresholds of less than 12 hours for warm ischemia and up to 24 hours for cold ischemia in digits [5]. However, emerging evidence suggests that favorable outcomes can still be achieved in selected cases of delayed replantation, especially when the amputated part is adequately preserved, and the injury is caused by a sharp mechanism [6].

Saw-cut injuries, which typically produce clean transections with minimal soft tissue damage, are associated with higher success rates compared to crush or avulsion injuries [4,6]. Furthermore, advancements in microsurgical techniques, perioperative care, and structured rehabilitation protocols have significantly improved survival rates and functional outcomes following digital replantation [7,8].

We present a case of delayed thumb replantation performed approximately 24 hours after injury in a young manual worker, demonstrating satisfactory functional and clinical outcomes despite prolonged ischemia time.

## Case presentation

A 25-year-old right-hand-dominant male, employed as a skilled worker in a wood-processing factory, presented to the emergency department with a traumatic amputation of the left thumb following a saw-cut injury sustained at work. The injury occurred approximately 24 hours prior to presentation. The amputated thumb segment had been appropriately preserved and transported with the patient. The patient had no significant past medical history and was otherwise healthy.

**Figure 1:**
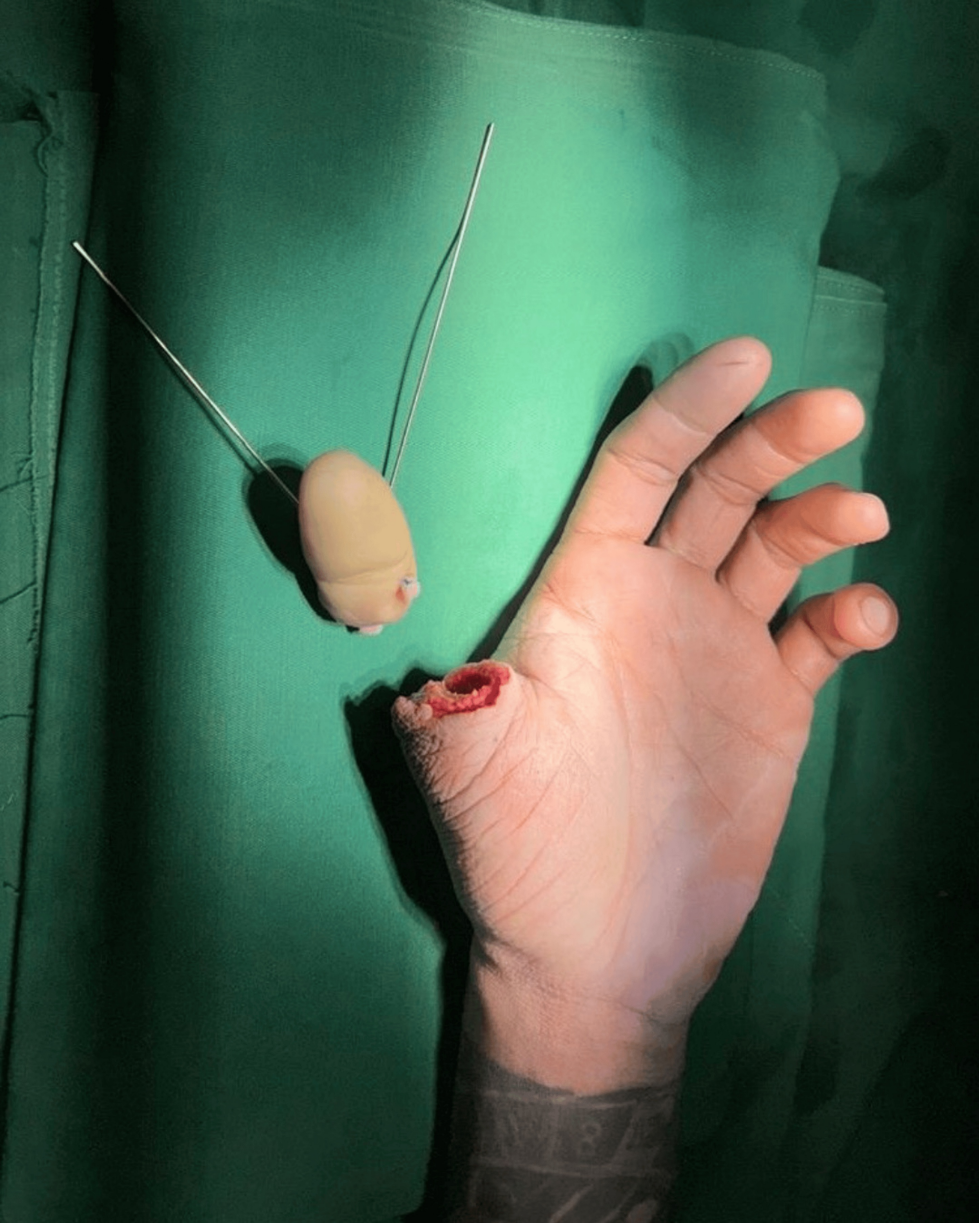
Initial presentation Clinical photograph showing traumatic amputation of the left thumb at the level of the proximal phalanx following a saw-cut injury. The amputated thumb segment is preserved and shown adjacent to the stump. Written informed consent was obtained from the patient for publication of this case report and accompanying images.

On clinical examination, there was a complete amputation of the left thumb at the level of the base of the proximal phalanx, with associated soft tissue injury but minimal contamination. The amputated thumb and stump at presentation are shown in Figure 1. The remaining digits were intact, with preserved vascularity and neurological function.

**Figure 2:**
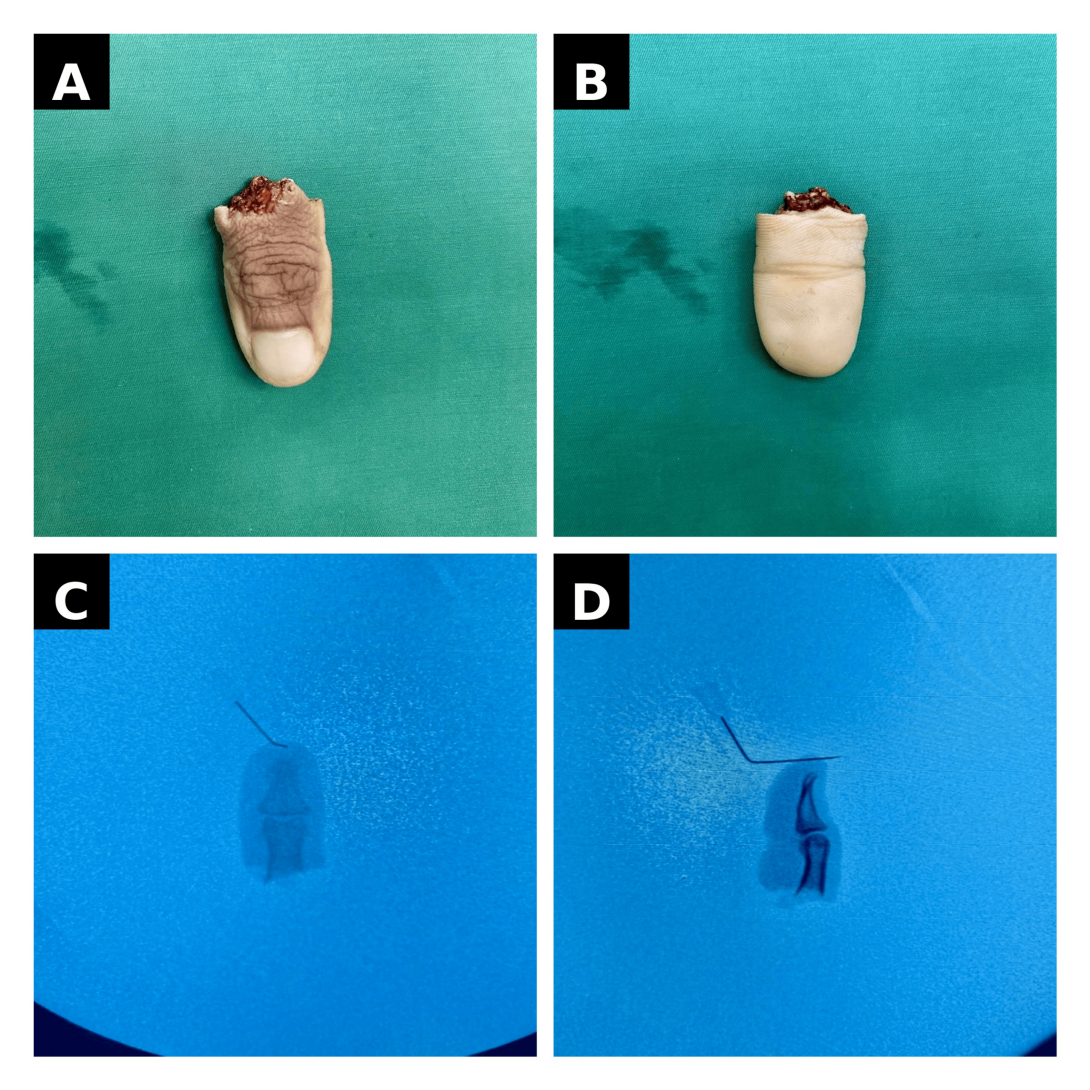
Gross and radiological evaluation of the amputated thumb (A) Dorsal view of the amputated segment. (B) Volar view of the amputated segment. (C) Fluoroscopic view showing bony alignment of the amputated segment. (D) Fluoroscopic lateral view demonstrating proximal phalanx fracture configuration.

On further assessment, the amputation was consistent with a clean-cut mechanism with minimal crush component. The radial digital artery was identified as the dominant arterial supply at the level of injury, and suitable dorsal veins were noted for potential venous repair. There was no significant contamination of the wound. Detailed evaluation of the amputated segment, including gross morphology and fluoroscopic imaging, is illustrated in Figure 2, confirming the level of bony injury and preserved structural integrity. Additional preoperative views of the injured hand demonstrate the extent of soft tissue damage and the clean transection pattern consistent with a saw-cut mechanism.

Given the patient’s young age, dominant hand involvement, and occupational requirement for fine hand function, a decision was made to proceed with thumb replantation despite the prolonged ischemia time.

The procedure was performed under regional anesthesia using a brachial plexus block. Following standard sterile preparation and draping, both the amputated segment and the proximal stump were thoroughly irrigated with normal saline and meticulously debrided to remove non-viable tissue while preserving all identifiable neurovascular structures. Minimal bone shortening was performed to facilitate tension-free repair, and skeletal stabilization was achieved using crossed Kirschner wires (K-wires) to align the proximal phalanx. Adequate alignment and stability were confirmed under fluoroscopic guidance.

Under magnification, the neurovascular bundles were identified and prepared. The digital artery, measuring approximately 1.0 mm in diameter, and the accompanying vein, measuring approximately 1.2 mm, were dissected and trimmed to healthy margins. End-to-end arterial and venous anastomoses were performed using interrupted 9-0 polypropylene (Prolene; Ethicon Inc., Somerville, NJ, USA) sutures, according to surgeon preference. Restoration of circulation was confirmed intraoperatively by immediate capillary refill, bleeding from the wound edges, and improvement in skin color.

**Figure 3:**
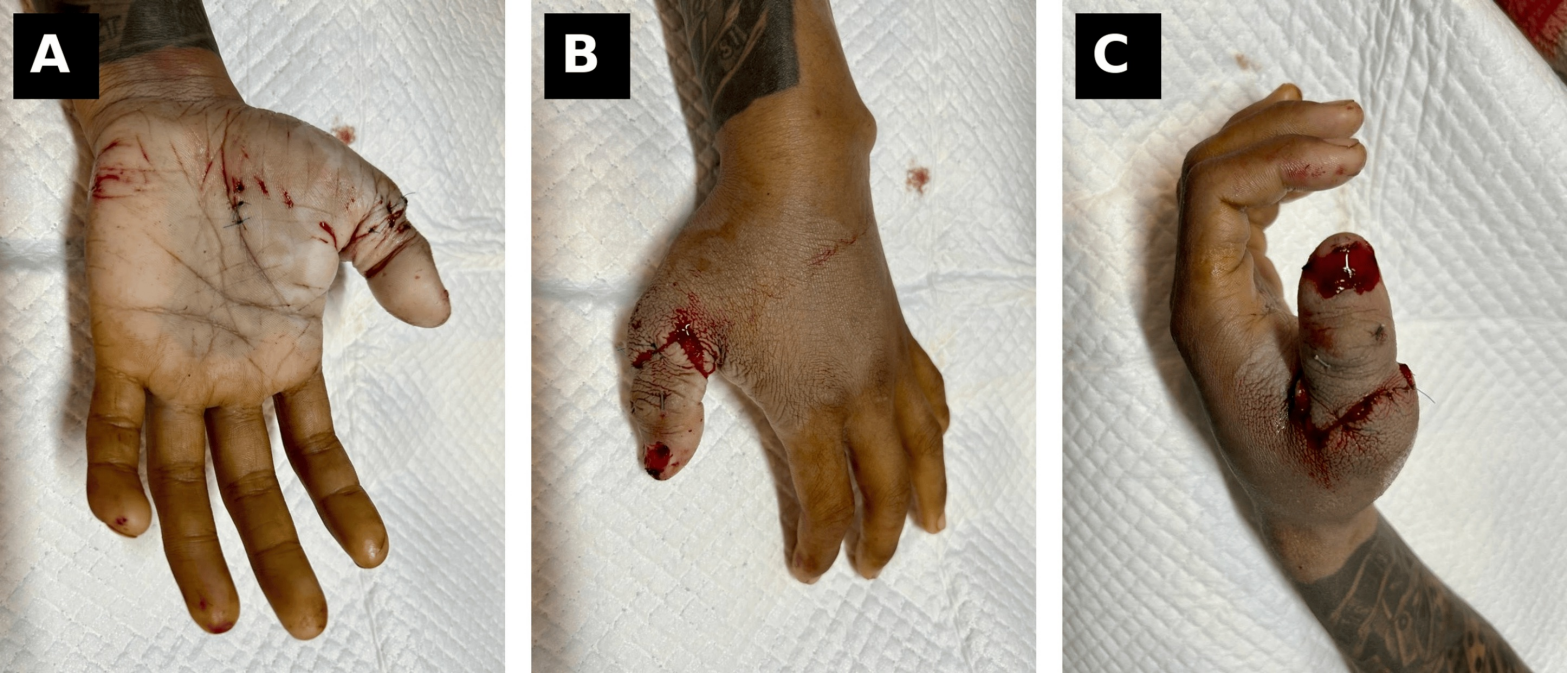
Immediate postoperative appearance of the replanted thumb (A) Volar view demonstrating stump at proximal phalanx level. (B) Dorsal view showing soft tissue injury and contamination. (C) Oblique view highlighting the level of amputation and soft tissue condition. Written informed consent was obtained from the patient for publication of this case report and accompanying images.

The digital nerve, measuring approximately 1.5 mm in diameter, was repaired using epineurial coaptation with interrupted 9-0 polypropylene sutures, ensuring proper alignment without tension to optimize sensory recovery. The extensor tendon was repaired using 4-0 polypropylene sutures with reinforcement using 5-0 polypropylene, while the flexor pollicis longus tendon was repaired using a core suture technique with 4-0 polypropylene and epitendinous reinforcement using 5-0 polypropylene. Skin closure was performed using 4-0 nylon sutures without tension, ensuring adequate soft tissue coverage. A sterile dressing and protective splint were applied to maintain the thumb in a functional position (Figure 3). The patient was started on intravenous cefazolin (1 g every eight hours) with additional metronidazole coverage due to the open nature of the injury. Postoperative anticoagulation included low-dose heparin infusion and antiplatelet therapy (aspirin 75 mg daily) to reduce the risk of microvascular thrombosis.

**Figure 4:**
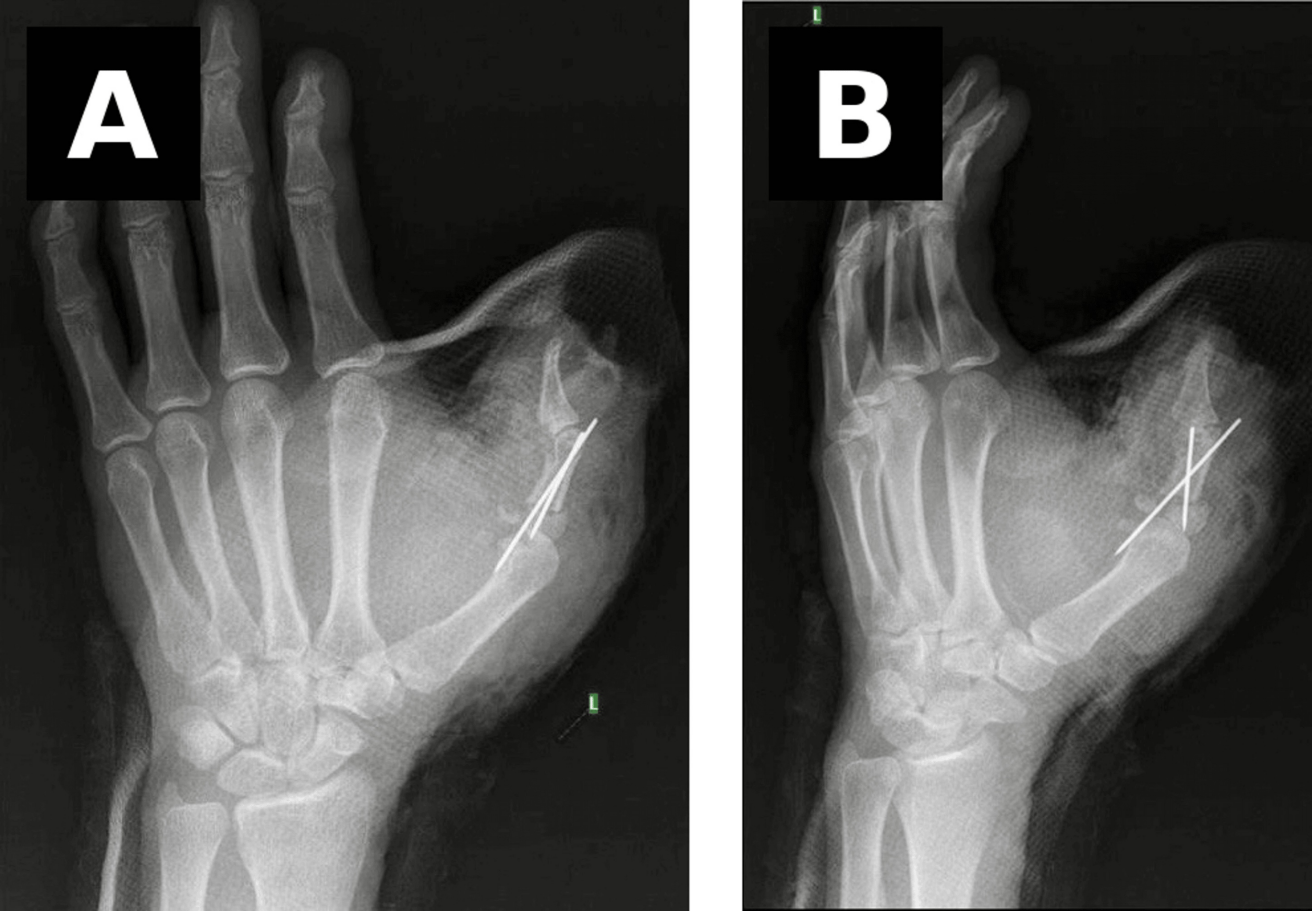
Postoperative radiographs following thumb replantation (A) Anteroposterior view demonstrating K-wire fixation of the proximal phalanx. (B) Lateral view confirming alignment and stability of the replanted thumb.

Immediate postoperative radiographs confirmed satisfactory alignment and stable fixation of the replanted thumb, as shown in Figure 4.

**Figure 5:**
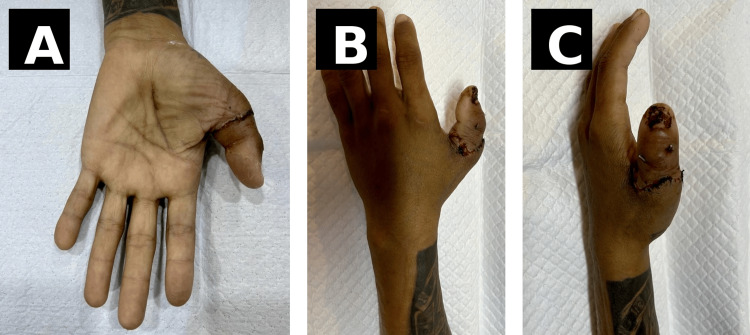
Six-week postoperative clinical outcomes following thumb replantation (A) Volar view showing viable skin and healing surgical wound. (B) Dorsal view demonstrating maintained thumb position and soft tissue healing. (C) Lateral view illustrating thumb alignment and soft tissue recovery. Written informed consent was obtained from the patient for publication of this case report and accompanying images.

Postoperatively, the patient was closely monitored for vascular viability and complications. Vascular status was closely monitored using clinical parameters, including capillary refill, skin color, temperature, and pinprick bleeding. No episodes of vascular compromise were observed during the postoperative period. A standardized anticoagulation protocol was initiated, consisting of low-molecular-weight heparin (enoxaparin 40 mg subcutaneously once daily) and oral aspirin 75 mg daily to reduce the risk of microvascular thrombosis. Adequate hydration, limb elevation, and maintenance of a warm environment were ensured to optimize perfusion. Early postoperative clinical appearance demonstrating satisfactory perfusion and soft tissue viability is shown in Figure 5. Serial clinical assessments revealed no evidence of vascular compromise or infection.

The patient was followed up weekly for the first four weeks, followed by assessments at six weeks, three months, and six months postoperatively. The K-wires were removed at six weeks postoperatively. Clinical and radiographic evidence of bone union was observed at approximately eight weeks, with maintained alignment and stability. A structured rehabilitation program was implemented, with initial immobilization for two weeks followed by gradual introduction of passive and active range-of-motion exercises. Strengthening exercises were initiated at approximately six weeks postoperatively, focusing on grip and pinch function, with progressive functional training thereafter.

**Figure 6:**
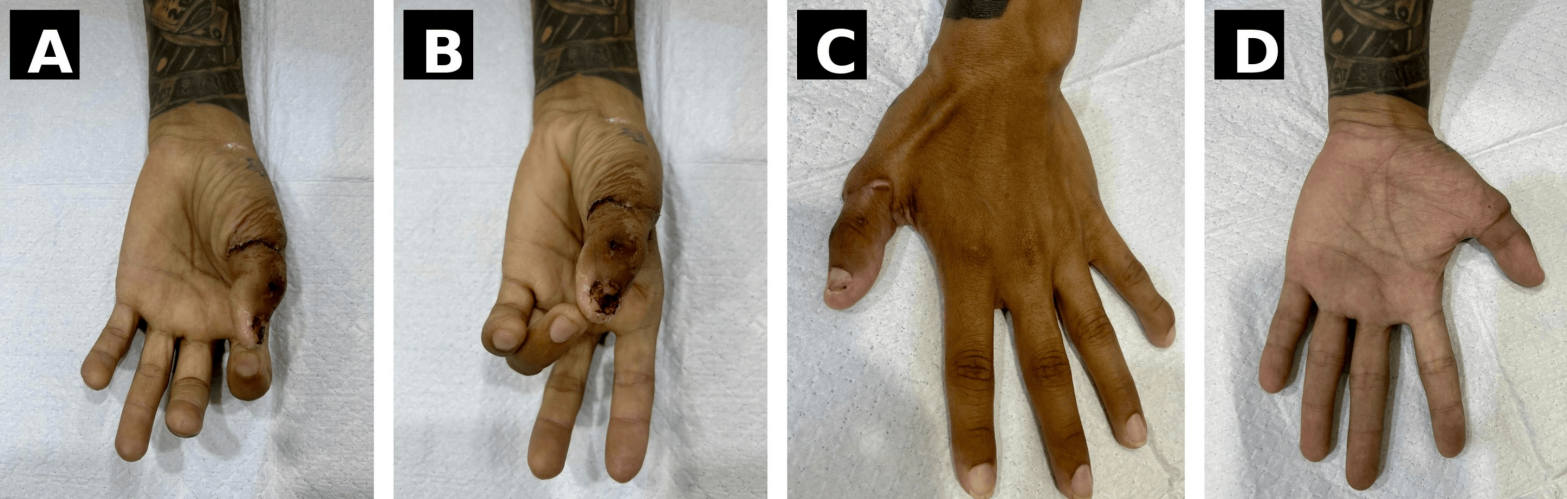
Six-month follow-up clinical outcomes demonstrating functional recovery. (A) Volar view showing healed surgical site. (B) Flexion posture demonstrating active thumb movement. (C) Dorsal view showing alignment and soft tissue healing. (D) Functional hand position illustrating restoration of grasp and opposition. Written informed consent was obtained from the patient for publication of this case report and accompanying images.

At the six-month follow-up, the patient demonstrated good functional outcomes. Grip strength, measured using a hand dynamometer, was approximately 38-42 kg, comparable to the contralateral side. Key pinch strength was approximately 6.5-7.5 kg, and tip pinch strength ranged between 4.5 and 5.5 kg. The patient achieved full thumb opposition, corresponding to a Kapandji score of 9-10. Functional range of motion at the metacarpophalangeal and interphalangeal joints was preserved. Sensory recovery was also assessed, demonstrating protective sensation with a static two-point discrimination of approximately 6-8 mm over the thumb pulp. Final follow-up images demonstrating functional recovery and hand position are shown in Figure 6. A functional hand position was achieved, with restoration of effective grasp, pinch, and thumb opposition, allowing the patient to return to activities of daily living and occupational tasks without significant limitation. The functional outcome of the replanted thumb, including active movement and opposition, is demonstrated in Video 1.

**Video 1:** Six-month follow-up clinical outcome A functional hand position was achieved, with restoration of effective grasp and thumb opposition. Written informed consent was obtained from the patient for publication of this case report and accompanying images/videos.

## Discussion

Thumb replantation remains a priority in hand trauma management due to its essential role in prehension, dexterity, and overall hand biomechanics [1,2]. The present case demonstrates that successful replantation can be achieved even after a prolonged ischemia time of approximately 24 hours, challenging traditional limitations associated with delayed presentation.

Ischemia time is a key determinant of replantation success; however, digits have a higher tolerance to ischemia compared to major limb segments due to the absence of muscle tissue, which is more susceptible to necrosis [5]. Adequate preservation of the amputated part, particularly under cold conditions, plays a crucial role in extending tissue viability and improving outcomes in delayed cases [3,5].

The mechanism of injury is another important factor influencing survival rates. Sharp injuries, such as those caused by saws, typically result in minimal vascular damage and well-defined tissue planes, facilitating microvascular repair and improving replantation success [4,6]. In contrast, crush and avulsion injuries are associated with extensive soft tissue damage and higher complication rates [3].

Functional recovery following thumb replantation depends on multiple factors, including stable skeletal fixation, tendon repair, nerve coaptation, and early rehabilitation [7,8]. In this case, the use of K-wire fixation provided adequate stabilization, allowing for proper alignment and healing. Structured rehabilitation contributed significantly to the restoration of function.

At six months postoperatively, the patient achieved near-normal grip strength (38-42 kg), key pinch strength (6.5-7.5 kg), and full opposition (Kapandji score 9-10), which are consistent with reported outcomes in successful replantation cases [9]. These findings emphasize that meaningful functional recovery can be achieved even in delayed replantation when appropriate surgical and rehabilitative strategies are employed.

Recent studies have also highlighted that replantation offers superior functional outcomes compared to revision amputation, particularly in cases involving the thumb [10]. Therefore, the decision to attempt replantation should not be based solely on ischemia time but should consider patient factors, injury characteristics, and available surgical expertise.

This case highlights several important clinical considerations in delayed thumb replantation. Although replantation is ideally performed within a shorter ischemia time, successful outcomes may still be achieved beyond conventional limits in selected cases. In our patient, the relatively clean-cut mechanism, proper preservation of the amputated segment, and absence of significant contamination were favorable factors contributing to success. In addition, meticulous intraoperative technique, particularly precise microvascular anastomosis and adequate venous outflow, played a critical role in maintaining tissue viability.

One of the key challenges encountered in this case was the prolonged ischemia time of approximately 24 hours, which increases the risk of endothelial damage and thrombosis. Careful intraoperative assessment of vessel quality and selection of a suitable artery and vein were essential to ensure successful revascularization. The use of a single arterial inflow and one dorsal venous outflow was sufficient in this case, demonstrating that complete anatomical restoration is not always required if physiological perfusion is achieved.

Furthermore, this case emphasizes the importance of comprehensive postoperative management, including close vascular monitoring and appropriate anticoagulation, to prevent complications such as venous congestion or arterial insufficiency. Early rehabilitation also contributed to the favorable functional outcome observed at follow-up.

Compared with previously reported cases of delayed replantation, including those with prolonged ischemia times, our findings support the concept that replantation should not be excluded solely based on time criteria. Instead, decision-making should consider injury characteristics, patient factors, and available surgical expertise. This case adds to the growing body of evidence suggesting that carefully selected patients can achieve good functional outcomes even after delayed replantation. This case provides practical insight into decision-making and technical considerations in delayed thumb replantation and may assist surgeons in managing similar challenging situations.

## Conclusions

Thumb replantation remains the optimal treatment for traumatic thumb amputation due to its critical role in hand function. Although prolonged ischemia time is traditionally considered a limiting factor, this case demonstrates that successful replantation can still be achieved even after a delay of approximately 24 hours when appropriate tissue preservation, careful patient selection, and meticulous microsurgical techniques are employed. Favorable functional outcomes, including restoration of grip strength, pinch strength, and opposition, can be obtained with structured postoperative rehabilitation. This case highlights the importance of considering replantation in delayed presentations, particularly in young, active individuals with high functional demands, rather than relying solely on conventional ischemia time thresholds.

## References

[1] Sebastin SJ, Chung KC (2011). A systematic review of the outcomes of replantation of distal digital amputation. Plast Reconstr Surg.

[2] Higgins RM (1991). Replantation of digits. Orthop Nurs.

[3] Friedrich JB, Poppler LH, Mack CD, Rivara FP, Levin LS, Klein MB (2011). Epidemiology of upper extremity replantation surgery in the United States. J Hand Surg Am.

[4] Renfro KN, Eckhoff MD, Trevizo GAG, Dunn JC (2024). Traumatic finger amputations: epidemiology and mechanism of injury, 2010-2019. Hand (N Y).

[5] Zhu H, Bao B, Zheng X (2018). A comparison of functional outcomes and therapeutic costs: single-digit replantation versus revision amputation. Plast Reconstr Surg.

[6] Chang J, Jones N (2004). Twelve simple maneuvers to optimize digital replantation and revascularization. Tech Hand Up Extrem Surg.

[7] Wei FC, Mardini S (2009). Flaps and Reconstructive Surgery. Plast Reconstr Surg.

[8] Tang JB (2021). Rehabilitation after flexor tendon repair and others: a safe and efficient protocol. J Hand Surg Eur Vol.

[9] Dec W (2006). A meta-analysis of success rates for digit replantation. Tech Hand Up Extrem Surg.

[10] Ruijs AC, Niehof SP, Selles RW, Jaquet JB, Daanen HA, Hovius SE (2009). Digital rewarming patterns after median and ulnar nerve injury. J Hand Surg Am.

